# Characteristics of Allergen Labelling and Precautionary Allergen Labelling in Packaged Food Products Available in Latin America

**DOI:** 10.3390/nu12092698

**Published:** 2020-09-04

**Authors:** Noé Ontiveros, Jesús Aristeo-López Gallardo, Jesús Gilberto Arámburo-Gálvez, Carlos Eduardo Beltrán-Cárdenas, Oscar Gerardo Figueroa-Salcido, José Antonio Mora-Melgem, Diana María Granda-Restrepo, Cecilia Ivonne Rodríguez-Bellegarrigue, Marcela de Jesús Vergara-Jiménez, Feliznando Isidro Cárdenas-Torres, Martina Hilda Gracia-Valenzuela, Francisco Cabrera-Chávez

**Affiliations:** 1 Department of Chemical, Biological, and Agricultural Sciences (DC-QB), Division of Sciences and Engineering, Clinical and Research Laboratory (LACIUS, URS), University of Sonora, Navojoa 85880, Sonora, Mexico; noeontiveros@gmail.com; 2 Faculty of Nutrition Sciences, University of Sinaloa, Culiacán 80019, Sinaloa, Mexico; aristeo.lopez37@hotmail.com (J.A.-L.G.); carlos.1.beltran@hotmail.com (C.E.B.-C.); joseantoniomoramelgem@gmail.com (J.A.M.-M.); mjvergara@uas.edu.mx (M.d.J.V.-J.); feliznando@uas.edu.mx (F.I.C.-T.); 3 Postgraduate in Health Sciences, Division of Biological and Health Sciences, University of Sonora, Hermosillo 83000, Sonora, Mexico; gilberto.aramburo.g@gmail.com (J.G.A.-G.); gerardofs95@hotmail.com (O.G.F.-S.); 4 Food Department, Faculty of Pharmaceutical and food sciences, University of Antioquia, Antioquia 50010, Medellín, Colombia; diana.granda@udea.edu.co; 5 Luis Edmundo Vasquez School of Health Sciences, Department of Public Health, Dr. José Matías Delgado University, Antiguo Cuscatlán 1502, El Salvador; cirodriguezb@ujmd.edu.sv; 6 Technological Institute of the Yaqui Valley, Bácum 82276, Sonora, Mexico

**Keywords:** allergen labelling, Latin America, packaged food products

## Abstract

The characteristics of food allergen labelling are relevant for avoiding accidental exposure to the allergens of interest but no Latin American country has evaluated these characteristics. Our aim was to evaluate the characteristics of food allergen labelling and precautionary allergen labelling (PAL) in six Latin American countries. All data were collected directly from the supermarkets surveyed. A total of 10,254 packaged food products were analyzed, of which 63.3% (*n* = 6494) and 33.2% (*n* = 3405) featured allergen labelling and/or PAL, respectively. Most products complied with local regulations (≥87.4% for both locally produced and imported). Thirty-three types of PAL statements were detected; the most frequent was “may contain traces of…” (35.1%). Countries without regulations on the characteristics of allergen labelling had two-fold more products that contained allergens in their ingredients lists but no food allergen labelling. The use of PAL in countries that regulate it (38.2%) was as high as that in countries without PAL regulations (19.2%–44.7%). The findings suggest that the lack of regulations for the characteristics of allergen labeling increases the risk of accidental exposure to allergens of interest. Our findings also suggest that beyond regulations, a scientific approach is required for minimizing and standardizing the use of PAL.

## 1. Introduction

Food-allergic individuals must avoid the allergen of interest in their diets. Food-allergic reactions can vary from mild symptoms to life-threatening anaphylaxis. Intergovernmental members of the Codex Alimentarius have established mandatory general guidelines for the allergen labelling of packaged food products and countries can adapt these guidelines to their particular needs and regulations [1,2]. Despite this, accidental exposure to the allergen of interest often occur, which sometimes have fatal consequences [3,4,5]. The prevalence rates of food allergies in children and adults vary between 0.6–10.5% and 3–10%, respectively, but current data suggest that the prevalence of this condition is increasing worldwide [6,7]. This fact represents a challenge for packaged food producers regarding the declaration of allergens. In this context, for allergens used as ingredients in the production of specific foods, allergen labelling must be clear and understandable for the general population and comply with local and overseas regulations for exportation purposes. Conversely, there are no regulations in most countries for packaged food products at risk of cross-contamination with food allergens during the manufacturing process and food producers often use precautionary allergen labelling (PAL) (i.e., “may contain” statements) for filling this gap [8]. Although PAL is not intended to replace allergen risk analysis and management, most food products with PAL do not have detectable levels of the allergen(s) of interest and, therefore, do not represent a risk for the vast majority of food-allergic individuals [9,10]. This fact encourages food-allergic individuals to take the risk of consuming foods with PAL and casts doubts among healthcare professionals about the safe use of foods with PAL in allergen-restricted diets [11,12,13]. Furthermore, the excessive use of PAL limits the availability of packaged food products for food-allergic individuals and/or parents/caregivers, which could give rise to an additional economic burden [9,12,13]. Developed and emerging countries have evaluated the prevalence and characteristics of food products with PAL [14,15,16] but in Latin America, no study has addressed either the characteristics of food allergen labelling or PAL. Regulations for food allergen labelling can vary from country to country, with the characteristics of the label and the list of allergens declared on it representing the main sources of variation [2]. The Latin American region has particular characteristics in trade, including food products. There are three major trade blocs in Latin America: the Common Market of the South (MERCOSUR) (encompassing Argentina, Brazil, Paraguay, Uruguay, and Venezuela), the Pacific Alliance (encompassing Chile, Colombia, México, and Peru), and the Secretariat for Central American Economic Integration (SIECA) (encompassing Costa Rica, Guatemala, Honduras, El Salvador, Nicaragua, and Panama) but the harmonization of food regulations remains debated [8,17]. Furthermore, although most Latin American countries regulate the allergens to be declared, the characteristics of food allergen labelling are not regulated in some countries such as México, Panamá, and Colombia, and only Argentina, Brazil, and Chile have regulated the use of PAL [17,18,19,20]. Thus, the aim of the present study was to evaluate the characteristics of food allergen labelling and PAL in six Latin American countries.

## 2. Materials and Methods

### 2.1. Survey and Data Collection

Cluster sampling was carried out from January to December 2019. Supermarkets were chosen based on information available online and the suggestions of local citizens. At least one local and one multinational supermarket featuring a flow of consumers with different socioeconomic statuses were included in the survey. Three supermarkets were sampled in Culiacán and three in Mexico City (México, North America), three in San Salvador (El Salvador), four in Panama City (Panamá, Central America), four in Medellín (Colombia), three in Quito (Ecuador), and four in Buenos Aires (Argentina, South America). The sample size consisted of all the packaged food products available on the shelves at the time of the survey. For products with multiple package sizes, only one size was included in the analysis to avoid bias [14]. Duplicate products found across the supermarkets in each country were recorded only once. Information was captured via digital images that covered the brands and name of the products as well as ingredients and details of allergen labelling, PAL (when available), and place of manufacture [15]. All images were verified twice to corroborate the match between the label captions and products. The information extracted from the images consisted of the following: product brand and name, place of manufacture, ingredients, food allergen declaration, typography (bold, italics, highlighted, colored, etc.), and PAL statements.

### 2.2. Food Categories and Allergens

Food products were categorized into twelve groups: baked goods, snacks, confectionaries, baby foods, condiments and sauces, jams and spreads, beverages, powders and pastes, instant foods, chilled and frozen foods, canned foods, and packaged raw foods [15]. The food allergens considered by the Codex Alimentarius guidelines were the focus of the present study (milk and its derivates, egg, soy, wheat, and other cereals containing gluten, peanuts, nuts, fish, and crustaceans) [1].

### 2.3. Compliance with the Regulations for the Characteristics of Food Allergen Labelling

Both local and imported packaged food products were verified for their compliance with local regulations for the characteristics of allergen labelling regulations or Codex Alimentarius guidelines for this purpose. The following characteristics were verified: for Argentina, allergen labelling statements with the legend “contains…” and the use of special typography (capital letters, bold letters, or different colors than the label) as well as the place where the allergen labelling statement appeared on the packaging [18]; for Ecuador, allergen labelling statements with the legend “contains…”, the use of capital letters, and the place where the allergen labelling statement appeared on the packaging [21]; for El Salvador, the allergen labelling statement with any legend, the use of any special typography to highlight the allergen labelling statement, and the place where the allergen labelling statement appeared on the packaging [22]; for Colombia, México, and Panamá, the allergen labelling statement with any allergen labelling statement and typography [1,23,24]. Regardless of whether the products featured food allergen labelling, all ingredient lists were inspected to verify that they did not include undeclared allergens, i.e., products without food allergen labelling but containing allergens as ingredients were also recorded. The analyses of compliance with local regulations for the characteristics of food allergen labelling only included products with food allergen labelling.

### 2.4. Frequency of Food Allergen Labelling, PAL, and the Type of PAL

All products were examined for corroborating the presence of either food-allergic labelling or PAL or both types of allergen labelling. Independently, if the food allergen was declared in the list of ingredients (or not), the products were examined for the presence of PAL. All types of PAL were included in the study.

### 2.5. Data Analysis

Descriptive statistics was used and the results are shown as percentages. Confidence intervals were calculated (95%) using the OpenEpi software version 3.01.

## 3. Results

### 3.1. Frequency of Food Allergen Labelling in Packaged Food Products

**Figure 1:**
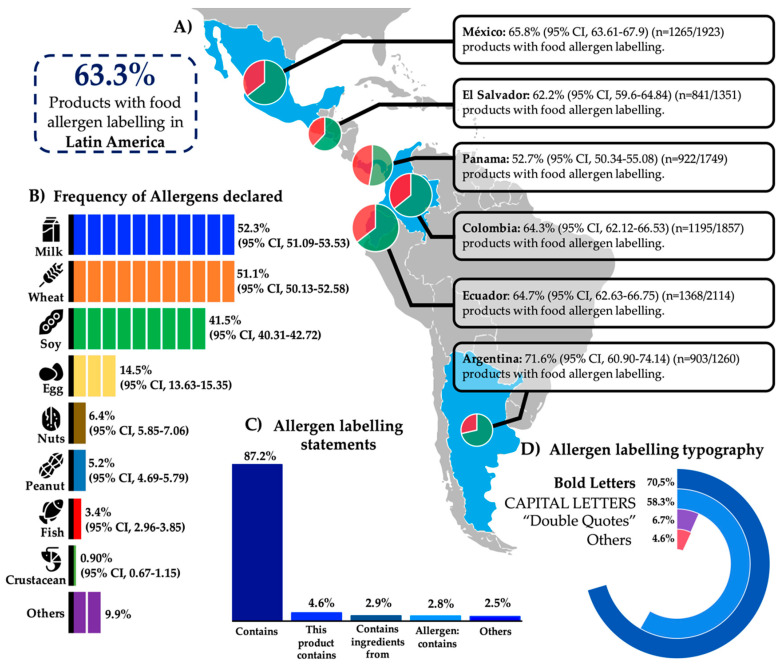
Percentage of products with food allergen labelling and the characteristics of the labelling in commercially available packaged food products in six Latin American countries. (**A**): Percentage of products with food allergen labelling by country (in pie charts: green and red sections; proportions of products with or without food allergen labelling, respectively); (**B**): Percentage of food allergens declared in food allergen labelling; (**C**): Statements used for food allergen labelling; (**D**): Typography used for food allergen labelling (the summation of percentages is greater than 100% due to the use of two or more typographical characteristics in combination).

Twenty-four supermarkets from six countries (seven cities) were sampled. In total, 10,254 packaged food product labels were analyzed: 71.1% of the products (*n* = 7288; 95% CI, 70.19–71.95) had food allergen labelling and/or PAL. Most of the products without food allergen labelling or PAL were naturally allergen-free products such as chips, juices, rice cookies, and packaged raw food among others (67.70%; *n* = 2008/2966; 95% CI, 65.98–69.38), while 63.3% (*n* = 6494; 95% CI, 62.39–64.26) of the products featured food allergen labelling (Figure 1). Panamá and Argentina had the lowest (*n* = 922; 52.71%) and the highest (*n* = 903; 71.66%) percentages of products with food allergen labelling, respectively (Figure 1A). The most commonly declared allergens were milk, including its derivatives (*n* = 3397; 52.32%); wheat and other cereals containing gluten (*n* = 3335; 51.36%); and soybean, including its derivatives (*n* = 2696; 41.52%) (Figure 1B). The less frequently declared food allergens were fish and crustaceans (*n* = 220; 3.38% and *n* = 58; 0.90%, respectively) (Figure 1B). The most frequent statements used for food allergen labelling were “contains…” (87.2%) followed by “this product contains…” (4.6%), “Allergens: contains…” (2.9%), and “contains ingredients from…” (2.8%) (Figure 1C). The most commonly used typography for allergen labelling statements was bold letters (70.52%), in either lower case or capital letters, and capital letters without bold typography (58.3%) (Figure 1D).

### 3.2. Compliance with Local Regulations for the Characteristics of Food Allergen Labelling

**Figure 2:**
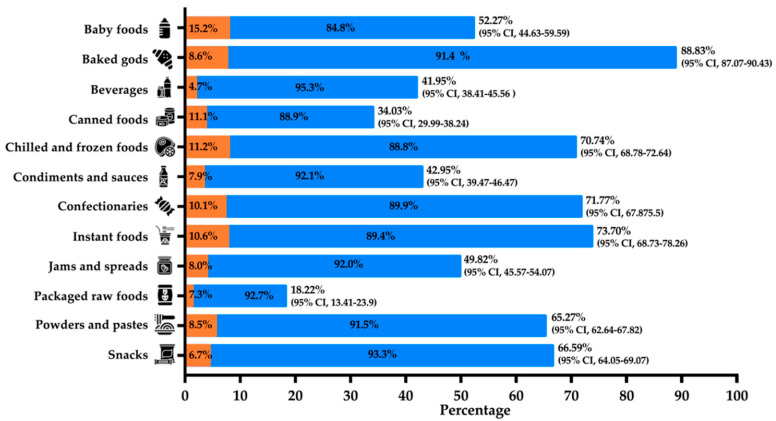
Compliance and non-compliance with local regulations for the characteristics of food allergen labelling by food category. Percentages in orange bars (█): frequency of non-compliance of food products with local regulations for food allergen labelling; Percentages in blue bars (█): frequency of products in compliance with local regulations for food allergen labelling. Percentages at the tops of the bars correspond to the percentages of packaged foods with food allergen labelling.

Most packaged foods with food allergen labelling were compliant with the local regulations for this purpose (91.0%, *n* = 5909/6494). Beverages (95.3%), snacks (93.1%), and packaged raw food (92.7%) were the food categories that featured the best compliance with local regulations (Figure 2). The category of baby foods was non-compliant with local regulations to a greater extent than the other categories (15.2%) and was one of the categories with the lowest percentage of allergen labelling (52.27%) (Figure 2). For local or imported food products, 90.67% (range: 77.44%–97.11%) and 87.44% (range: 74.39%–97.92%) of locally produced and imported products complied with local regulations, respectively (Table 1). In total, 6494 products had food allergen labelling and 585 of them (9.0%) were in non-compliance with local regulations. Of these 585 products, 263 (45.0%) had undisclosed allergens in their food allergen labelling, 295 (50.4%) did not use the proper allergen labelling typography, and 76 products (13.0%) used an allergen labelling statement other than those enforced by local regulations (Supplementary Material Table S1). El Salvador, Ecuador, and Argentina were the countries with the highest proportions of products in non-compliance with local regulations (22.00%, 11.47%, and 8.30%, respectively), followed by México, Colombia, and Panamá (6.24%, 5.77%, and 2.16%, respectively) (Table 1). Countries with the highest proportions of products without food allergen labelling but containing allergens as ingredients were Panamá (36.15%, *n* = 299), Colombia (35.64%, *n* = 236), and México (35.10%, *n* = 231), followed by El Salvador (32.74%, *n* = 167), Argentina (22.12%, *n* = 79), and Ecuador (15.81%, *n* = 118) (Table 1). The number of products without food allergen labelling but containing allergens as ingredients was more than two-fold higher, on average, in Colombia, Panamá, and México than in Argentina, Ecuador, and El Salvador (766 vs. 364, respectively) (Table 1). On average, this means that for every 5.7 (1130 out of 6494) products with allergen labelling, there is one product without food allergen labelling but containing allergens as an ingredient in the Latin American countries included in the survey (Table 1).

### 3.3. Frequency and Characteristics of PAL

**Figure 3:**
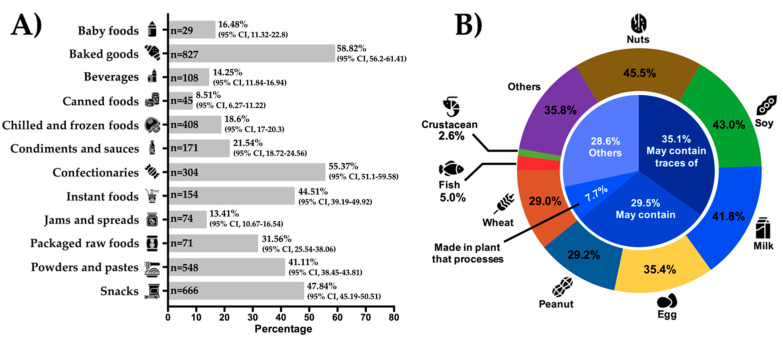
Percentages of packaged foods with precautionary allergen labelling (PAL) and the most frequent types of PAL. (**A**): frequencies of products with PAL by food categories; (**B**): pie center: most frequently used types of PAL in packaged foods; pie periphery: most frequently declared food allergens in PAL (The summation of percentages is more than 100% due to a combination of two or more allergens in PAL).

The proportion of products with PAL was 33.2% (*n* = 3405/10,254; 95% CI, 32.29–34.13). This accounts for 46.7% of the total of products with food allergen labelling and/or PAL (3405/7288). PAL was identified in 58.8% (*n* = 827) of the baked goods, 55.37% (*n* = 304) of confectionaries, and 47.84% (*n* = 666) of snacks (Figure 3A). PAL was identified in only 8.5% (*n* = 45) of the canned foods category (Figure 3A). The most frequently used PAL statements were “may contain traces of…” (*n*= 1195; 35.01%), “may contain…” (*n* = 1004; 29.5%), and “made in plant that processes…” (*n* = 261; 7.7%) (Figure 3B). Thirty more PAL statements were identified in 28.60% (*n* = 974) of the products evaluated (Table S1). Some products declared two different types of PAL (0.9%; *n* = 31). The most commonly declared allergens in PAL were nuts (*n* = 1549; 45.5%), soy (*n* = 1466; 43%), and milk (*n* = 1423; 41.8%) (Figure 3B). On average, PAL was identified in 23.6% (*n* = 622) of naturally allergen-free food products and in 25.5% (*n* = 2611) of products with food allergen labelling. Ecuador (19.2%; *n* = 407), Panamá (24.9%; *n* = 436), and El Salvador (31.7%; *n* = 428) had the lowest percentages of products with PAL and Argentina (38.2; *n* = 482), México (42.7%; *n* = 822), and Colombia (44.7%; *n* = 482) had the highest percentages.

## 4. Discussion

The correct allergen labelling of food products is essential to minimize accidental exposure to the allergens of interest among food-allergic individuals and to ameliorate the socioeconomic burden associated with this condition. In the present study, the characteristics of the food allergen labelling and PAL of products available in six Latin American countries were analyzed. The results show that, independently of the country sampled, at least one in two packaged food products available on the shelves of the main supermarkets featured food allergen labelling. This illustrates that food-allergic individuals should analyze the labels of each packaged food product that they choose to buy for the first time or analyze such labels regularly to ensure that food producers have not changed their ingredients or manufacturing processes. Consequently, food-allergic individuals will spend additional time buying foods at the supermarket because they have to identify suitable foods. This extra time can be 39% greater than the time spent by non-food-allergic individuals [9]. The most commonly declared food allergens in the six Latin American counties surveyed were the eight foods considered for allergen labelling in the Codex Alimentarius guidelines (milk, wheat, soy, egg, nuts, peanuts, fish, and crustaceans) [1]. These major food allergens declared in the allergen labelling analyzed were reported to be the main triggers of immediate-type food allergy symptoms by the parents of 11,277 Latin American children with different nationalities [25,26,27,28]. Certainly, as per the opinions of others [17], the scientific evidence is still insufficient to state that food allergens other than those considered by the Codex Alimentarius guidelines should be added to the food allergen labelling regulations in Latin American countries. The method for stating food allergens in allergen labelling is important to avoid misinterpretations and to easily identify the presence of the allergen of interest. In the present study, the most common allergen labelling statements were “contains”, “this product contains”, “contains ingredients from”, and “allergen: contains”, either in bold or capital letters or a combination of these typographies. These results are consistent with the current regulations of Argentina and Ecuador, which enforce the use of the statement “contains” in capital letters (Ecuador) or special typography (capital letters, bold letters, or different colors than the label (Argentina) [18,21]. Although previous studies highlighted that using symbols in combination with word-based allergen labelling statements can be more effective for communicating with food-allergic individuals than using word-based labelling only [29,30], no Latin American regulations for food allergen labelling enforce the use of symbols for such a purpose. The use of symbols in food allergen labelling could, perhaps, reduce the extra time that food-allergic individuals spend identifying suitable foods.

Regulations for food allergen labelling are mainly intended to protect food-allergic individuals from accidental exposure to the allergen of interest and establish the characteristics of the labels. Non-compliance with local regulations entails sanctions ranging from financial penalties to partial or total closure of the responsible company. In general, 91.0% of the food allergen labelling analyzed complied with local regulations. The food category of baked goods had the highest percentage of allergen labelling (88.8%) and was also one of the food categories with the highest and lowest percentages of compliance (91.4%) and non-compliance (8.6%) with local regulations for allergen labelling. Wheat is the major allergenic ingredient in most conventional formulations of baked goods so its declaration in food allergen labelling is mandatory across all Latin American countries [17]. Furthermore, wheat-based baked goods trigger symptoms not only in wheat-allergic individuals but also in some individuals with celiac disease or irritable bowel syndrome as well as in all those with non-celiac gluten sensitivity [31]. Conversely, baby food was one of the categories with the lowest percentage of allergen labelling (52.3%). Most of the baby foods without allergen labelling had no allergens in their ingredient lists but 6.52% of them had undisclosed allergens. This is intriguing because the target population of these foods is particularly vulnerable for developing food allergies [31]. Although food allergen labelling regulations can vary from one country to another, on average, the percentages of locally produced (90.7%) and imported (87.4%) packaged foods that complied with local food allergen labelling regulations were quite similar. This suggests that beyond differences in country to country regulations, most packaged food products comply with the local food allergen labelling regulations where they are sold. Argentina, Ecuador, and El Salvador established the typography to be used for the food allergen labelling statement and the place where the statement has to be located on the food packaging, with differences in typography between Ecuador and the other two countries [18,21,22]. Furthermore, Argentina and Ecuador have regulated the use of “contains” as their allergen labelling statement [18,22]. Colombia and México have also established food allergen labelling regulations but the allergen labelling statement, typography of the statement, and place where the statement has to be located on the food packaging remain to be established [23,24]. Panamá has not yet established regulations for food labelling [17] but the Codex Alimentarius guidelines are enforced [32,33]. As expected, the countries with “weak” allergen labelling regulations (Colombia, México, and Panamá) had the highest percentage (92.2%, on average) of packaged food products that complied with local allergen labelling regulations. Although this compliance could facilitate the trading of foods among countries, it could also have serious consequences for food-allergic individuals. In this context, our findings show that some products without allergen labelling contained allergens as ingredients and that, on average, the number of products with this peculiarity was two-fold higher in countries that have not yet established the characteristics for their food allergen labelling statements compared with those that have already done so (766 vs. 364). Thus, the risk of choosing a food product at the supermarket without allergen labelling when that food contains allergens is two-fold higher in countries that have not yet regulated the characteristics for food allergen labelling. Others have reported undisclosed allergens in packaged food products using analytical methods [34,35,36]. Overall, our results suggest that regulations should consider all the characteristics of food allergen labelling to avoid putting food-allergic individuals at increased risk of accidental exposure to their allergens of interest.

PAL is used in food products at the risk of cross-contamination with food allergens during the manufacturing process but there are no regulations for its use in most countries. The present study shows that PAL is widely used in Latin America (33.2% of food products) and that even the proportion of naturally allergen-free products with PAL is considerable (23.6%). Others have reported similar percentages of PAL (28.6%–39.9%) [14,15,37] or even higher (65%) [38]. The PAL statement “may contain traces of…” was the most commonly utilized statement, which agrees with other studies [15,38] but 32 other types of PAL were also identified in the present study. It should be noted that PAL generates anxiety and confusion among food-allergic individuals and/or parents/caregivers [39] as its excessive use could contribute to increasing the socioeconomic disease burden [12]. Furthermore, both healthcare professionals and food-allergic individuals are facing a dilemma regarding what to do with PAL products [11,12,13] as most food products with PAL have undetectable levels of allergens and are not risky for most food-allergic individuals [9,10]. Argentina had one of the highest percentage of products with PAL (38.2%) although this country was also the only one that has regulated the use of PAL among the countries surveyed [9,18]. These findings suggest that the regulation of PAL may not be enough to limit its use or to ameliorate the negative impact that the excessive use of PAL has on food-allergic individuals and/or their parents/caregivers. In addition to Argentina, other countries such as South Africa, Brazil, and Chile have regulated the use of PAL. Legislation in these countries allows the use of PAL as long as its use is substantiated by a documented risk assessment demonstrating adherence to good manufacturing practices [9,19,20]. Alternatively, a scientific approach called voluntary incidental trace allergen labelling (VITAL) was developed by the Australian manufacturing industry in 2007 [39]. This approach is based on the reference doses (thresholds) for specific allergens [12,40]. An allergen dose that is likely to trigger allergic reactions in 1% or 5% of the food-allergic population is called ED01 or ED05, respectively [12]. This promising proposal has not been yet endorsed by public health agencies [12] but it seems to have the approval of the majority of the scientific community. Undoubtedly, the endorsement of VITAL by public health agencies, its regulation by countries, and its implementation by food producers will strongly contribute to ameliorating the socioeconomic burden of food allergies and solving the dilemma on how to govern foods products with PAL.

The main strengths of the study are its large number of labels analyzed and its inclusion of countries with different regulations for the characteristics of food allergen labelling including a country that has regulated the use of PAL. These strengths allowed us to draw deeper conclusions than previous studies that have been carried out in only one country. The main limitations of this study include not addressing consumer understandings, perceptions, and interpretations of food allergen labelling and the lack of laboratory tests for confirming the presence or absence of food allergens in products with PAL or in products without food allergen labelling but containing allergens as ingredients. Despite these limitations, the present study provides groundwork for future studies based on immunological methods for detecting food allergens in packaged food products and increases our knowledge about the characteristics of food allergen labelling in the Latin American region.

## 5. Conclusions

This is the first study carried out in Latin America to evaluate the characteristics of food allergen labelling in commercially available packaged foods. The packaged food products available with food allergen labelling accounted for 63.3% and the vast majority of those products, both locally produced and imported, complied with local regulations. The countries (Colombia, México, and Panamá) that have not yet regulated the characteristics of their food allergen labelling had more than two-fold greater number of products without labelling when those products contained allergens as ingredients compared with the countries that have already done so (Argentina, Ecuador, and El Salvador), putting their food-allergic populations at increased risk of accidental exposure to their allergens of interest. The use of PAL was high in all of the countries surveyed including one country that has regulated the use of PAL. Furthermore, there were many types of PAL (33), with the most common being “may contain traces of…”. Additional strategies for PAL regulations should be implemented to minimize and standardize the use of PAL.

## Figures and Tables

**Table 1:** Compliance and non-compliance with local regulations for the characteristics of food allergen labelling.

		Argentina (*n* = 1260)	Colombia (*n* = 1857)	Ecuador (*n* = 2114)	El Salvador (*n* = 1351)	México (*n* = 1923)	Panamá (*n* = 1749)	Total (*n* = 10,254)
Food allergen labelling	% (*n*)	71.66 (903)	64.35 (1195)	64.71 (1368)	62.25 (841)	65.78 (1265)	52.71 (922)	63.33 (6494)
	95% CI	69.12–74.09	62.15–66.5	62.65–66.72	59.63–64.8	63.63–67.87	50.37–55.05	62.39–64.26
Compliance with the local regulations	% (*n*)	91.69 (828)	94.22 (1126)	88.52 (1211)	78.0 (656)	93.75 (1186)	97.83 (902)	90.99 (5909)
	95% CI	89.71–93.32	92.76–95.41	86.73–90.11	75.08–80.67	92.28–94.96	96.67–98.59	90.27–91.66
Non-compliance with the local regulations	% (*n*)	8.30 (75)	5.77 (69)	11.47 (157)	22.0 (185)	6.24 (79)	2.16 (20)	9.01 (585)
	95% CI	6.67–10.29	4.58–7.24	9.89–13.27	19.33–24.92	5.04–7.71	1.40–3.32	8.33–9.72
No Food allergen labelling	% (*n*)	28.33 (357)	35.64 (662)	35.28 (746)	37.74 (510)	34.21 (658)	47.28 (827)	36.66 (3760)
	95% CI	25.91–30.88	33.50–37.85	33.28–37.35	35.20–40.37	32.13.36.37	44.95–49.63	35.74–37.61
No Food allergen labelling but allergens in ingredient list	% (*n*)	22.12 (79)	35.64 (236)	15.81 (118)	32.74 (167)	35.10 (231)	36.15 (299)	30.05 (1130)
	95% CI	18.13–26.72	32.09–39.37	13.38–18.61	28.81–36.93	31.56–38.83	32.95–39.49	28.61–31.54

## Supplementary Materials

The following are available online at https://www.mdpi.com/2072-6643/12/9/2698/s1, Table S1: Variations of precautionary allergen labelling (PAL).
