# Supplementary material for: Characteristics of Allergen Labelling and Precautionary Allergen Labelling in Packaged Food Products Available in Latin America
Source: Nutrients. 2020 Sep 4;12(9):2698. doi: 10.3390/nu12092698 (PMC7576488; doi:10.3390/nu12092698)
Supplement: Supplementary file 1 [file nutrients-12-02698-s001.docx]

**Table S1.** Variations of precautory allergen labelling (PAL).

| **Variations of precautory allergen labelling** | **n** | **%** | **CI 95%** |
| --- | --- | --- | --- |
| May contain traces of [allergen(s)]^1^ | 1195 | 35.10 | 33.49-36.73 |
| May contain [allergen(s)]^2^ | 1004 | 29.49 | 27.96-31.05 |
| Made in a plant that processes [allergen(s)]^3^ | 261 | 7.67 | 6.79-8.61 |
| Made in equipment that processes products that contain [allergen(s)]^4^ | 158 | 4.64 | 3.95-5.40 |
| Made in teams that process [allergen(s)]^5^ | 142 | 4.17 | 3.52-4.89 |
| Made in equipment that processes products with [allergen(s)]^6^ | 142 | 4.17 | 3.52-4.89 |
| This product may contain traces of [allergen(s)]^7^ | 81 | 2.38 | 189-2.94 |
| Made online where it is processed [allergen(s)]^8^ | 73 | 2.14 | 1.68-2.68 |
| Made in a plant that processes products containing [allergen(s)]^9^ | 46 | 1.35 | 0.99-1.79 |
| This product was made in equipment that also processes [allergen(s)]^10^ | 37 | 1.09 | 0.76-1.49 |
| Packed in facilities that handle [allergen(s)]^11^ | 29 | 0.85 | 0.57-1.22 |
| Packaged in a factory that processes [allergen(s)]^12^ | 27 | 0.79 | 0.52-1.15 |
| Manufactured in teams that make products with [allergen(s)]^13^ | 24 | 0.70 | 0.45-1.04 |
| Product manufactured in equipment that processes [allergen(s)]^14^ | 24 | 0.70 | 0.45-1.04 |
| This product is made in the same facilities where products containing [allergen(s)] are handled^15^ | 22 | 0.65 | 0.40-0.87 |
| This product is made in equipment that processes [allergen(s)]^16^ | 21 | 0.62 | 0.38-0.94 |
| This product is made in a food plant where other ingredients are used and may contain traces of [allergen(s)]^17^ | 17 | 0.50 | 0.29-0.79 |
| This product may contain [allergen(s)]^18^ | 16 | 0.47 | 0.26-0.76 |
| May contain ingredients of [allergen(s)]^19^ | 16 | 0.47 | 0.26-0.76 |
| Made in equipment that processes food containing [allergen(s)]^20^ | 16 | 0.47 | 0.26-0.76 |
| This product is manufactured in equipment where products with [allergen(s)] are processed^21^ | 15 | 0.44 | 0.24-0.72 |
| Made in machinery where [allergen(s)] are processed^22^ | 10 | 0.29 | 0.14.0.53 |
| Made in a factory that packs [allergen(s)]^23^ | 10 | 0.29 | 0.14.0.53 |
| This product may contain residues of [allergen(s)]^24^ | 8 | 0.23 | 0.10-0.46 |
| This product is manufactured in facilities and equipment where products that may contain [allergen (s)] are also processed^25^ | 7 | 0.21 | 0.08-0.42 |
| This product is made from ingredients that had contact with equipment that processes [allergen (s)]^26^ | 6 | 0.18 | 0.06-0.38 |
| Processed in machines that process [allergen(s)]^27^ | 6 | 0.18 | 0.06-0.38 |
| Produced in a factory that uses [allergen(s)]^28^ | 6 | 0.18 | 0.06-0.38 |
| This product is made in the same facilities where products containing [allergen (s)] are handled^29^ | 4 | 0.12 | 0.03-0.30 |
| Made in the same production line in which other products are made that may contain traces of [allergen(s)]^30^ | 4 | 0.12 | 0.03-0.30 |
| Made in lines of [allergen(s)]^31^ | 3 | 0.09 | 0.01-0.25 |
| The [ingredient] used in this product may contain [allergen(s)]^32^ | 2 | 0.06 | 0.006-0.21 |
| This product has been produced on production lines where derivatives of [allergen (s)] have been processed^33^ | 2 | 0.06 | 0.006-0.21 |

**Continue table S1.**

| **Variations of PAL translated into spanish language** |
| --- |
| ^1^Puede contener trazas de [alérgeno(s)] |
| ^2^Puede contener [alérgeno(s)] |
| ^3^Elaborado en una planta que procesa [alérgeno(s)] |
| ^4^Elaborado en equipo que procesa productos que contienen [alérgeno(s)] |
| ^5^Elaborado en equipos que procesan [alérgeno(s)] |
| ^6^Elaborado en equipo que procesa productos con [alérgeno(s)] |
| ^7^Este producto puede contener trazas de [alérgeno(s)] |
| ^8^Elaborado en linea donde se procesa [alérgeno(s)] |
| ^9^Elaborado en una planta que procesa productos que contienen [alérgeno(s)] |
| ^10^Este producto fue elaborado en equipo que también procesa [alérgeno(s)] |
| ^11^Empacado en instalaciones que manejan [alérgeno(s)] |
| ^12^Envasado en una fabrica que procesa [alérgeno(s)] |
| ^13^Fabricado en equipos que elaboran productos con [alérgeno(s)] |
| ^14^Producto manufacturado en equipo que procesa [alérgeno(s)] |
| ^15^Este producto es elaborado en instalaciones que procesan productos con [alérgeno(s)] |
| ^16^Este producto se elabora en equipo que procesa [alérgeno(s)] |
| ^17^Este producto es elaborado en una planta de alimentos donde se usan otros ingredientes y puede contener trazas de [alérgeno(s)] |
| ^18^Este producto puede contener [alérgeno(s)] |
| ^19^Puede contener ingredientes de [alérgeno(s)] |
| ^20^Fabricado en equipos que procesan alimentos que contienen [alérgeno(s)] |
| ^21^Este producto es manufacturado en equipo donde se procesan productos con [alérgeno(s)] |
| ^22^Elaborado en maquinaria donde se procesan [alérgeno(s)] |
| ^23^Elaborado en una fabrica que empaca [alérgeno(s)] |
| ^24^Este producto puede contener residuos de [alérgeno(s)] |
| ^25^Este producto se fabrica en instalaciones y equipo donde tambien se procesan productos que pueden contener [alérgeno(s)] |
| ^26^Este producto se elabora con ingredientes que pudieron haber tenido contacto con equipos que procesan [alérgeno(s)] |
| ^27^Procesado en maquinas que procesan [alérgeno(s)] |
| ^28^Producido en una fabrica que utiliza [alérgeno(s)] |
| ^29^Este producto es elaborado en las mismas instalaciones donde se manejan productos que contienen [alérgeno(s)] |
| ^30^Elaborado en la misma linea de producción en la cual se elaboran otros productos que pueden contener trazas de [alérgeno(s)] |
| ^31^Elaborado en lineas de [alérgeno(s)] |
| ^32^El [ingrediente] utilizado en este producto puede contener trazas de [alérgeno(s)] |
| ^33^Este producto ha sido elaborado en lineas de producción donde se han procesado derivados de [alérgeno(s)] |
